# Cellular Senescence in Neurodegenerative Diseases

**DOI:** 10.3389/fncel.2020.00016

**Published:** 2020-02-11

**Authors:** Carmen Martínez-Cué, Noemí Rueda

**Affiliations:** Department of Physiology and Pharmacology, Faculty of Medicine, University of Cantabria, Santander, Spain

**Keywords:** senescence, Alzheimer’s disease, Down syndrome, Parkinsion’s disease, neurodegenaration

## Abstract

Cellular senescence is a homeostatic biological process characterized by a permanent state of cell cycle arrest that can contribute to the decline of the regenerative potential and function of tissues. The increased presence of senescent cells in different neurodegenerative diseases suggests the contribution of senescence in the pathophysiology of these disorders. Although several factors can induce senescence, DNA damage, oxidative stress, neuroinflammation, and altered proteostasis have been shown to play a role in its onset. Oxidative stress contributes to accelerated aging and cognitive dysfunction stages affecting neurogenesis, neuronal differentiation, connectivity, and survival. During later life stages, it is implicated in the progression of cognitive decline, synapse loss, and neuronal degeneration. Also, neuroinflammation exacerbates oxidative stress, synaptic dysfunction, and neuronal death through the harmful effects of pro-inflammatory cytokines on cell proliferation and maturation. Both oxidative stress and neuroinflammation can induce DNA damage and alterations in DNA repair that, in turn, can exacerbate them. Another important feature associated with senescence is altered proteostasis. Because of the disruption in the function and balance of the proteome, senescence can modify the proper synthesis, folding, quality control, and degradation rate of proteins producing, in some diseases, misfolded proteins or aggregation of abnormal proteins. There is an extensive body of literature that associates cellular senescence with several neurodegenerative disorders including Alzheimer’s disease (AD), Down syndrome (DS), and Parkinson’s disease (PD). This review summarizes the evidence of the shared neuropathological events in these neurodegenerative diseases and the implication of cellular senescence in their onset or aggravation. Understanding the role that cellular senescence plays in them could help to develop new therapeutic strategies.

## Introduction

Cellular senescence, a homeostatic process that reduces proliferation and helps to prevent the propagation of damaged cells (Vicencio et al., 2008; Coppé et al., 2010; Faragher et al., 2017; Yanagi et al., 2017), has been proposed to be a type of cell differentiation (Stein et al., 1991). Among its most relevant biological functions are the counteraction of uncontrolled cell proliferation, which avoids the formation of tumors, and the facilitation of the elimination of cells that are damaged or that are no longer necessary (Kültz, 2005). Cellular senescence has an essential physiological role during development (Muñoz-Espín and Serrano, 2014; Barbouti et al., 2019).

According to its duration and characteristics, senescence has been categorized into acute or chronic senescence (van Deursen, 2014). Acute senescence is implicated in normal biological processes during embryonic development or tissue repair. While, chronic senescence, induced by prolonged exposure to stress (Salama et al., 2014; van Deursen, 2014; Childs et al., 2015; Velarde and Menon, 2016; Watanabe et al., 2017; Ogrodnik et al., 2019), produces cellular and tissue alterations. Although both types of senescent cells display similar characteristics *in vitro* and *in vivo*, they are triggered by different stimuli and have differential consequences on the tissue.

Chronic cellular senescence contributes to functional alterations associated with aging and neurodegenerative diseases (Howcroft et al., 2013); the number of cells with this phenotype increases with healthy aging (Herbig et al., 2004; Lawless et al., 2010).

Different categories of chronic senescence have been identified, including replicative senescence, stress-induced premature senescence (SIPS), and mitochondrial dysfunction-associated senescence, among others (Kuilman et al., 2010).

Replicative senescence is the process in which human fibroblasts cultured *in vitro* end up displaying the hallmarks of senescence (a rise in protein p21 levels cell cycle arrest, SA-β-Gal activation, and morphological alterations; Romanov et al., 2012; Sanders et al., 2013). Replicative senescence is currently considered a model of aging (Chen et al., 2007).

Different types of cells of the central nervous system (CNS) can become senescent, including astrocytes (Pertusa et al., 2007; Mansour et al., 2008; Salminen et al., 2011), microglia (Evans et al., 2003; Flanary and Streit, 2004; Flanary et al., 2007; Bitto et al., 2010), oligodendrocytes (Al-Mashhadi et al., 2015), neurons (Sedelnikova et al., 2004; Jurk et al., 2012), and Neural Stem Cells (NSCs; Ferrón et al., 2004; He et al., 2013; Li et al., 2016). Senescence of these cell types have been implicated in the etiopathology of several neurodegenerative diseases (Streit et al., 2004, 2009; Conde and Streit, 2006; Baker et al., 2011; Bhat et al., 2012; He et al., 2013; Nasrabady et al., 2018; Ohashi et al., 2018), including Alzheimer’s disease (AD), Parkinson Disease (PD), frontotemporal dementia, amyotrophic lateral sclerosis, and multiple sclerosis (Yurov et al., 2014; Biron-Shental et al., 2015). This review summarizes the main findings on the role of cellular senescence in the neurodegenerative diseases AD, Down syndrome (DS), and PD.

### Cellular Senescence: Phenotype, Triggering Mechanisms and Implication in Neurodegenerative Diseases

Senescent cells are characterized by:

(i) Permanent cell cycle arrest, due to the blockade to the entrance to the S phase of the cycle (Stein et al., 1999; Krenning et al., 2014).

(ii) Senescence-associated secretory phenotype (SASP), which consists of the synthesis and release of proinflammatory chemokines, cytokines, growth factors and metalloproteinases (Coppé et al., 2010; Acosta et al., 2013; Chen et al., 2015) responsible of neuroinflammatory processes (Coppé et al., 2010; Freund et al., 2010; Özcan et al., 2016).

During aging and in neurodegenerative diseases, microglial cells display altered morphology characteristic of senescence (Flanary and Streit, 2004; Streit et al., 2004; Flanary et al., 2007). However, it has to be taken into account that increased release of pro-inflammatory cytokines due to microglia or astroglia activation is not necessarily related to cellular senescence. During healthy aging, the brain suffers mild chronic inflammation (Yankner et al., 2008). There is evidence that this state is caused by the dysregulation of microglial activation (Sheng et al., 1998; Frank et al., 2006; von Bernhardi, 2007; Mosher and Wyss-Coray, 2014; von Bernhardi et al., 2015a), which enhances the release of numerous proinflammatory cytokines, including IL1α, IL-2, IL-6, IL-8, IL-12, IL-15, IL-17, IL-18, IL-22, IL-23, IL6, IFN-γ and TNFα (Sheng et al., 1998; Njie et al., 2012; von Bernhardi et al., 2015b; Minciullo et al., 2016; Ventura et al., 2017; Rea et al., 2018). Inflammation induces a higher release of proinflammatory cytokines in old than in young cells (Combrinck et al., 2002; Cunningham et al., 2005; Sierra et al., 2007; Henry et al., 2009).

In neurodegenerative diseases, neuronal damage also dysregulates microglia activation and induces an increase in the release of pro-inflammatory mediators (von Bernhardi, 2007; López-Otín et al., 2013).

(iii) Altered mitochondrial function and morphology, which is one of the most important triggers of senescence, mainly through the induction of oxidative stress, which will alter cellular signaling and SASP (Takahashi et al., 2005; Passos et al., 2007, 2010; Correia-Melo and Passos, 2015).

High levels of oxidative stress have been demonstrated to induce cellular senescence during aging (De Haan et al., 1996), promoting neuronal DNA damage (Chow and Herrup, 2015), a deregulated DDR (Sedelnikova et al., 2004), an alteration in the entry and progression of the cell cycle and changes in cell morphology (Monti et al., 1992), premature replicative senescence and an accelerated rate of telomere attrition per cellular replication (von Zglinicki et al., 2000; Serra et al., 2003).

Mitochondria is a major site of production as well as a target of reactive oxygen species (ROS), and its endogenous antioxidant pathways are essential to maintain physiological redox signaling (Brand, 2014). Furthermore, mitochondrial ATP production is necessary for neural activity. But aging deteriorates mitochondrial integrity and function leading to reduced ATP production, to enhanced ROS formation, mutations in mitochondrial DNA (Richter et al., 1988; Shigenaga et al., 1994), facilitating neurodegeneration (Dröge and Schipper, 2007).

Finally, mitochondrial ROS can also induce telomere attrition, and dysfunction (Passos et al., 2007) and mitochondrial dysfunction can also facilitate senescence by inducing alterations in the cell metabolism (Ziegler et al., 2015; Liguori et al., 2018).

(iv) Changes in cellular metabolism, partially due to the altered mitochondrial function, producing an increase in lysosomal senescence-associated-β-galactosidase (Lee et al., 2006; Weichhart, 2018), accumulation of lipofuscin in the cytoplasm (Georgakopoulou et al., 2013; Höhn and Grune, 2013), and a reduction in fatty acid synthesis (Wu et al., 2017). Due to the high energy demand, and increased oxidative stress the cells can accumulate high levels of DNA damage. This damage increases during normal aging, when the DNA repair capacity is reduced (Maynard et al., 2015).

(v) DNA damage that alters chromatin structure and activates the DNA damage response (DDR) (Nakamura et al., 2008; Rodier et al., 2011).

### Telomere Attrition and Telomere Dysfunction

During replicative senescence, telomeres, DNA–proteins that protect chromosome ends from degradation, and inappropriate recombinations or fusions shorten with every cell division (Blackburn, 1991; Zakian, 1995; Chan and Blackburn, 2004; Hockemeyer et al., 2005). Telomere shortening reduces the protection of the ends of the chromosomes and leaves them exposed to DNA damage that can be similar to DNA double-strand break (d’Adda di Fagagna et al., 2003; Takai et al., 2003; Herbig et al., 2004; Rodier et al., 2009, 2011) that triggers the DDR. During senescence, the increase in DDR leaves these cells unable to perform DNA repair (Galbiati et al., 2017). Under these circumstances, the cell stops dividing (Harley et al., 1990; Harley, 1991; de Lange, 2002; d’Adda di Fagagna et al., 2003) leading to the cell cycle arrest characteristic of senescence.

Telomere shortening has been proposed to be a major mechanism in aging, and age-related pathology and a marker of cellular senescence (Greider and Blackburn, 1987; Olovnikov, 1996; Bernatdotte et al., 2016). The role of telomere attrition in aging and its correlation with senescence has been demonstrated in studies in primates, mice and humans (Blasco et al., 1997; Lee et al., 1998; Herbig et al., 2006; Hewitt et al., 2012; Kong et al., 2013; López-Otín et al., 2013; Birch et al., 2015).

Besides cellular replication, several stimuli can affect telomeres. There is evidence that mitochondrial dysfunction induces telomere damage, and that telomere damage can produce mitochondrial alterations (Zheng et al., 2019). Besides, oxidative stress accelerates telomere damage producing senescence (von Zglinicki et al., 2000; von Zglinicki, 2002; Saretzki et al., 2003; Serra et al., 2003), and the intracellular oxidative stress of a cell correlates with telomere attrition and with its replicative potential (Richter and von Zglinicki, 2007).

Although, as mentioned above, many studies have shown a relation between telomere attrition, senescence and aging (Harley et al., 1990; Allsopp et al., 1992; Hao et al., 2005; Heidinger et al., 2012; Kaul et al., 2012; Reichert et al., 2013), telomere length does not always correlate with senescence (Karlseder et al., 2002; Stewart et al., 2003).

Several reports failed to associate telomere length and mortality risk in elder humans (Bischoff et al., 2006; Li et al., 2015). Also, some studies have demonstrated that telomere dysfunction is not dependent on its length. In mice, and baboons longer telomeres with DNA-damage have been associated with aging (Fumagalli et al., 2012; Hewitt et al., 2012; Jurk et al., 2014).

Finally, as previously mentioned, telomere dysfunction is not only related to senescence in healthy aging but it is also found in patients with dementia (Kota et al., 2015), AD (Cai et al., 2013), and PD (Maeda et al., 2012).

### Non-telomeric DNA Damage

#### Genomic Instability and DNA Damage and Repair

The continuous activation of DDR produces senescence in neurons (Fielder et al., 2017). DNA damage induces senescence, enhanced oxidative stress and an associated increase in β-galactosidase activity in neurons of old mice (Jurk et al., 2012). Because the efficiency of DNA repair decreases with age, and more complex, and less efficient DNA repair mechanisms are used, the increased DNA damage can lead to neurodegeneration (Vaidya et al., 2014).

(vi) Epigenetic modifications. Epigenomic changes in senescent cells include an imbalance in repressive and active histone marks, heterochromatic alterations including formation of senescence-associated heterochromatic foci (SAHF), histone variants, altered nucleosomal composition, alterations in DNA methylation pattern, alterations in nuclear lamina-chromatin interactions and on 3D genome organization that contributes to cellular dysfunction (Yang and Sen, 2018; Wagner, 2019). Micro-RNAs, also participate in the regulation of cellular senescence (Komseli et al., 2018); resistance to apoptotic death, controlled by the p53 and p21 stress response pathway (Tang et al., 2006).

Epigenetic modification such as histone modifications and specific DNA methylation changes, including alterations in the activity of methylation enzymes (Vanyushin et al., 1973), occur during aging and are associated with the neuropathology and the progression of various neurodegenerative diseases (Berson et al., 2018; Prasad and Jho, 2019; Wagner, 2019). During aging, these changes in DNA methylation and histone modifications alter chromatin architecture (Tsurumi and Li, 2012). Aged tissues also display alterations in the expression of some micro RNAs (Cencioni et al., 2013).

(vii) Morphological changes. Senescent cells display changes in the organization of nuclear lamina that modify nuclear morphology and gene expression (Freund et al., 2012). Due to cytoskeletal rearrangements, senescent cells display an increased size, a flat and irregular shape and changes in cell membrane composition (Ohno-Iwashita et al., 2010; Druelle et al., 2016; Cormenier et al., 2018).

(viii) Altered proteostasis. Senescence cells display increased unfolded protein response (UPR) associated with endoplasmic reticulum (ER) stress, which participates in the increase in ER size and the changes in the shape and size of these cells (Ohno-Iwashita et al., 2010; Cormenier et al., 2018).

Proteostasis refers to the balance and correct function of the proteome and requires proper synthesis, folding, quality control, and degradation rate of proteins. In eukaryotic cells, it depends on the accurate regulation of the proteasome, on the lysosomal system and autophagy, an intracellular degradation system of damaged proteins (López-Otín et al., 2013). Senescent cells lose protein homeostasis due to nucleolar dysfunction, autophagy and lysosomal anomalies and alterations in UPR indicative of ER stress. Correct proteostasis reduces the secretion of inflammatory cytokines (Tanaka and Matsuda, 2014), while its alteration (including protein misfolding aggregation and deposition) are a hallmark of many neurodegenerative diseases (see below for PD and AD).

**Figure 1:**
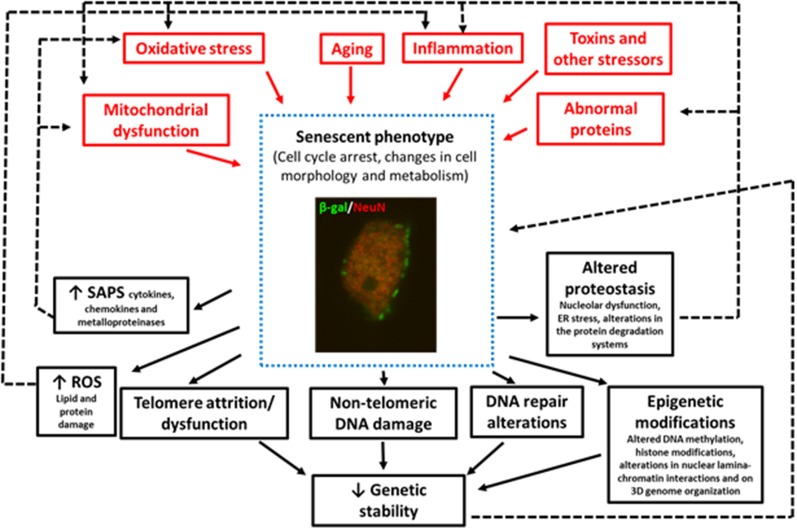
Image of a granular neuron (NeuN+) of the hippocampus of an aged wildtype mouse showing senescent phenotypes SA-β-Gal expression, and changes in cell morphology. This figure summarizes the triggering stimuli (in red) and the consequences (in black) of the increase in the number of cells with senescent phenotypes in neurodegenerative diseases. The black dotted lines represent the positive feedback mechanism that aggravates aging and neurodegeneration.

In summary, some of the factors that trigger senescence in pathological conditions are oxidative SIPS (Hernandez-Segura et al., 2018), mitochondrial dysfunction (Wiley et al., 2017), DNA-damage (Dörr et al., 2013), telomere attrition or dysfunction (Hayflick and Moorhead, 1961), aberrant gene activation (Gorgoulis and Halazonetis, 2010), epigenetic modifications (Yang and Sen, 2018; Wagner, 2019), and impaired autophagy (Kang et al., 2011), although other stressors can also induce senescence (Figure 1).

Finally, as previously mentioned, there is evidence that cellular senescence contributes to the pathogenesis of different neurodegenerative diseases through various mechanisms (Figure 1) including:

(i) The promotion of chronic inflammation: (Coppé et al., 2010). Thus, neuroinflammation is not only a trigger but also a result of senescence that can perpetuate the damage to the cell or neighboring cells (Nelson et al., 2012; Acosta et al., 2013; Ribezzo et al., 2016).

(ii) The promotion of oxidative stress and mitochondrial dysfunction. There is a positive feedback mechanism between senescence, oxidative stress, and mitochondrial dysfunction (Pole et al., 2016; Figure 1).

(iii) The reduction of the regenerative capacities of the nervous system: adult neurogenesis has been demonstrated in rodents (Kriegstein and Alvarez-Buylla, 2009; Ming and Song, 2011) and humans (Cipriani et al., 2018). Importantly, it has been recently demonstrated that neurogenesis is reduced in aged individuals and AD patients (Moreno-Jiménez et al., 2019). Due to the cell cycle arrest associated with cellular senescence, the reduced regenerative capacities of the brain (i.e., neurogenesis), would facilitate neurodegeneration.

(iv) Loss of function: finally, cell-cycle arrest and other alterations associated with cellular senescence alter the function of neurons and different CNS cell types (Purcell et al., 2014).

Aging increases the number of senescent cells (Rodier and Campisi, 2011) and the loss of neurons (De Stefano et al., 2016), compromising brain function and triggering or aggravating a neurodegenerative disease. Because senescent cells cannot maintain tissue function or repair its damage, if this state is maintained chronically, cellular aging and degenerative diseases would be aggravated.

In this review, we summarize the data that demonstrate the role of cellular senescence in the neurodegenerative diseases AD, DS, and PD (Table 1). Despite the differences in pathology among these three diseases, they are characterized by senescence and progressive loss of neurons leading to functional alterations (Nussbaum and Ellis, 2003).

**Table 1:** Summary of senescent phenotypes in normal aging, Alzheimer’s disease (AD), Down syndrome (DS), Parkinson Disease (PD).

Senescence phenotypes	Normal aging	AD	DS	PD
Cell cycle arrest	Cell cycle arrest (alterations in cell and tissue functions).	Cell cycle prolongation and re-entry.	Cell cycle arrest and elongation.	Cell cycle arrest (alterations in the expression of several genes implicated in the cell cycle).
SASP	Brain mild chronic inflammation, changes in microglia morphology, altered microglia activation, enhanced release of proinflammatory mediators.	Microglia overactivation, enhanced release of proinflammatory cytokines and other SASP that aggravate amyloid and tau pathology.	Microglia overactivation, proinflammatory mediators’ over-expression, increased release of proinflammatory cytokines from early late stages, as the person ages this is enhanced and aggravates AD neuropathology.	Activation of microglia and pro-inflammatory mediators release that has a role in the dopaminergic loss.
Oxidative stress and mitochondrial dysfunction	Increased ROS and mtROS. Altered mitochondrial integrity and function that compromises cell metabolism (reduces ATP) and induces damage DNA.	Increased ROS and altered mitochondrial structure and function that produces cellular changes associated with senescence.	Increased ROS and mtROS from embryonic stages. Altered mitochondrial structure and function that compromises cell metabolism (reduces ATP production). As the person ages, oxidative stress is enhanced and aggravates AD neuropathology. Oxidative stress-induced damage of DNA, lipids, and proteins.	Increased ROS and mtROS. Altered mitochondrial structure and function. Mutation of genes associated with pathways of mitochondrial dysfunction.
Telomeric DNA damage	Telomere attrition (replicative senescence) and damage.	Telomeric DNA damage. Regarding telomere shortening: controversial results.	Shorter telomeres correlate with the degree of dementia in DS individuals with AD.	Contradictory results regarding telomere length.
Non-telomeric DNA damage and DNA repair mechanism	Increased DNA damage accumulation and alteration in DDR.	Increased DNA damage and alterations in DDR.	Oxidative stress and other stressors enhance DNA damage accumulation. Overexpression of the USP16 gene and the reduced DNA POLb alter DNA repair mechanisms and chromatin remodeling. Genomic instability.	Genetic instability due to gene mutations, altered gene expression or regulation leading to cell cycle alterations. Impaired DNA repair mechanisms that increase the duration of cell cycle.
Epigenetic modifications	Alterations in histones, DNA methylation pattern, chromatin architecture, and micro RNAs expression.	Aberrant phosphorylation of histones, changes in DNA methylation of AD critical genes. Mislocated chromatin organizing proteins and epigenetic regulators.	Histone modifications, DNA hypermethylation, alteration in small non-coding RNAs implicated in premature aging and cognitive defects.	Changes in DNA methylation, posttranscriptional modification of histones.
Morphological changes	Increased size, flat and irregular shape, changes in membrane composition.	Increased size, flat and irregular shape, changes in membrane composition.	Increased size, flat and irregular shape, changes in membrane composition.	Increased size, flat and irregular shape, changes in membrane composition.
Altered proteostasis	Nucleolar dysfunction, altered transcription, endoplasmic reticulum stress, proteasome and autophagy anomalies.	Loss of protein homeostasis. Nucleolar dysfunction. Accumulation of abnormal proteins (amyloid peptides and hyperphosphorylated tau).	Increased gene copy number alters proteostasis. Alterations in the ribosome biogenesis machinery (increases number of nuclear organizer regions resulting in excessive rRNA and ribosomal protein synthesis leading to waste energy. Alterations in the protein degradative systems (autophagy and proteasome). Altered proteostasis leads to increases in amyloid aggregates and tau hyperphosphorylation.	Accumulation of misfolded proteins. Accumulation of neurotoxic α-synuclein associated with Lewy bodies. Autophagic/lysosomal dysfunction.
Others	Accumulation of lipofuscin		Early aging, and development of senescence phenotype in the brain and other tissues.	

## Cellular Senescence in Alzheimer’s Disease

AD is a chronic neurodegenerative disease that accounts for 60–70% of cases of dementia. The main pathological hallmarks of AD are dementia and cognitive impairment, amyloid plaques, neurofibrillary tangles (NFTs) of hyperphosphorylated tau proteins, and loss of neurons and synapses (Selkoe and Hardy, 2016). However, there is increasing evidence that some pathological events that appear years earlier than the former have a prominent role in the development of amyloid plaques and NFTs. These events include increased oxidative stress, neuroinflammation, and cellular senescence due to DNA damage and altered proteostasis. Numerous reports have demonstrated their role in the increase in Aβ burden, tau hyperphosphorylation, neuronal death and an accelerated cognitive decline (Hardy and Higgins, 1992; Gitter et al., 1995; Chong, 1997; Weldon et al., 1998; Eikelenboom et al., 2006; Hardy, 2006; Sipos et al., 2007; Wilcock, 2012).

Cellular senescence has been demonstrated to play an important role in the onset and aggravation of AD (Bhat et al., 2012; Boccardi et al., 2015). Increased senescence is found in different cell types of AD brains, including astrocytes, microglia, and neurons as demonstrated by their enhanced SA-β-gal expression (He et al., 2013), p53 expression, a mediator of cellular senescence (Arendt et al., 1996; McShea et al., 1997; Luth et al., 2000; Yates et al., 2015), an increase in the release of SASP components (Erusalimsky, 2009), DNA damage (Myung et al., 2008), telomere attrition or damage (Flanary and Streit, 2004), and senescence-like morphological changes (Streit et al., 2004).

Increased levels of senescent cells with higher SA-β-gal and p53 levels are also found and in plasma samples from AD patients and mouse models of AD (de la Monte et al., 1997; Tiribuzi et al., 2011; Magini et al., 2015; Caldeira et al., 2017).

Several studies have demonstrated an association between senescence and AD neurodegeneration. In cultured neurons, the expression of several senescent-associated genes was upregulated after the exposure to Aβ (Wei et al., 2016). These results were also confirmed *in vivo*, using the 5× FAD mouse model of AD that displays progressive Aβ deposition. After 7 months of age, this model shows the upregulation of some essential senescent—related genes in the hippocampus (Wei et al., 2016). Besides, a murine model that overexpresses human tau and develop NFT deposition also shows increased expression of several senescent—associated genes in the hippocampus and cortex (Bussian et al., 2018).

Besides, the administration of Aβ oligomers to oligodendrocyte progenitor cells can also induce senescence (Zhang et al., 2019) and in murine neural stem cells, Aβ42 peptides increase the number of SA-β-Gal positive cells (He et al., 2013). The removal of senescent cells in mice reduces Aβ accumulation and enhances their cognitive abilities (Zhang et al., 2019). Besides, hyperphosphorylation of tau can induce senescence in glial cells (Musi et al., 2018).

The fact that many of the early alterations found in AD (i.e., neuroinflammation, oxidative stress, DNA damage and changes in DNA repair and altered proteostasis) trigger or are associated with cellular senescence, has led to the suggestion that this process has a crucial role in the etiopathology of AD.

### Neuroinflammation

The brains of AD patients and of mouse models of AD have higher levels of inflammation due to microglia activation that produces pro-inflammatory cytokines and other SASP mediators (Streit et al., 2004; Hickman et al., 2008; Rawji et al., 2016; Olivieri et al., 2018). Neuroinflammation in the brain of AD patients is influenced by many SASPs mediators (Bauer et al., 1991; Huell et al., 1995; Kiecolt-Glaser et al., 2003) that have a prominent role in the onset of senescence (Flanary and Streit, 2004; Flanary et al., 2007). IL-6, a proinflammatory cytokine, is upregulated in the aged brain and AD (Bauer et al., 1991; Huell et al., 1995; Kiecolt-Glaser et al., 2003) and its overexpression has been shown to induce neurodegeneration (Campbell et al., 1993). The brains and lymphocytes of AD patients and AD mouse models also present increased activity of the SASP regulator p38MAPK (Sun et al., 2003). This enhanced p38MAPK activity upregulates the levels of the pro-inflammatory cytokines IL-6, IL-1, TGF-β and TNF-α levels in AD brains (Bauer et al., 1991; Huell et al., 1995; Freund et al., 2012; Lai et al., 2017; Rea et al., 2018), CSF and serum (Wood et al., 1993; Cacabelos et al., 1994; Blum-Degen et al., 1995; Luterman et al., 2000; Swardfager et al., 2010; Tarkowski et al., 2003; Gezen-Ak et al., 2013; Dursun et al., 2015). AD brains also present increased levels of other SASPs mediators: the metalloproteinases MMP-1, MMP-3, and MMP-10 (Leake et al., 2000; Yoshiyama et al., 2000; Bjerke et al., 2011; Horstmann et al., 2018). The fact that SASP meditators have a prominent role in the onset of senescence (Flanary and Streit, 2004; Flanary et al., 2007), provide support for the relationship between neuroinflammation and senescence in AD neuropathology.

Neuroinflammation aggravates AD (Guerreiro et al., 2013) mainly because it increases APP and Aβ expression in this disease. Pro-inflammatory cytokines induce the formation of Aβ oligomers, the phosphorylation of tau, and ROS production (Sastre et al., 2003; Blurton-Jones and Laferla, 2006; Steele et al., 2007). In turn, Aβ peptides and APP activate glial cells (Dickson et al., 1993; Barger and Harmon, 1997), produce an enhanced release of pro-inflammatory mediators such as IL-1 and IFNγ in the brains of AD patients (Ho et al., 2005; Meager, 2004, 2005). Under these circumstances, in AD, microglia internalize less Aβ (Floden and Combs, 2011; Njie et al., 2012) and are less able to process it (Nixon et al., 2001; Hickman et al., 2008; Mawuenyega et al., 2010). The higher release of pro-inflammatory cytokines reduces the ability of the cells to remove Aβ and facilitates its accumulation. Thus, there is a positive feedback between cytokine release, Aβ, and APP expression, phosphorylated tau and neurodegeneration (Wilcock and Griffin, 2013).

### Oxidative Stress and Mitochondrial Dysfunction

Oxidative stress is another crucial mechanism that contributes to accelerating aging and cognitive dysfunction in AD and during aging (López-Otín et al., 2013). AD patients and mouse models of AD, present increased oxidative stress and mitochondrial dysfunction, similar to that observed in senescence. These events are present in the early stages of AD and precede the major pathologic hallmarks, such as senile plaques and NFTs (Yates et al., 2015; Ott et al., 2018).

In AD mitochondrial function and structure are impaired (Cadonic et al., 2016; Tai et al., 2017), which has a critical role in the progression of SIPS (Gao et al., 2017). Besides, the enhanced production of ROS decreases ATP synthesis. Because DNA repair is a mechanism that requires a high amount of energy, and DNA is heavily damaged in cells exposed to oxidative stress (Monti et al., 1992), these cells would need a high amount of energy to perform DNA repair. Thus, mitochondrial dysfunction reduces the ability of the cell to repair these alterations.

As mentioned in the “Introduction” section, during aging, high levels of oxidative stress induce in cells a state of senescence that displays its main pathological characteristics, including DNA damage, altered DDR, alterations in the cell cycle and cell morphology and telomere damage (Monti et al., 1992; De Haan et al., 1996; von Zglinicki et al., 2000; Serra et al., 2003; Chow and Herrup, 2015; Kawanishi and Oikawa, 2004; Jennings et al., 2000; Liu et al., 2003). Thus, a link between oxidative stress and cellular senescence has been postulated in AD.

Evidence for the relation between oxidative stress and cellular senescence in AD comes from studies in mouse models of AD in which the animals were subjected to chronic oxidative stress (SIPS) and found cellular changes identical to those found in other types of senescence: i.e., an increase in SA-β-gal expression, cell cycle arrest and alterations in cellular morphology (Toussaint et al., 2000; Ma et al., 2014). Besides, fibroblasts from AD patients produced more ROS, a slowing in the growth rate, an increase in the expression of p53 and p21 and senescence-like phenotype (Toussaint et al., 2000; Naderi et al., 2006).

### DNA Damage and Repair

As mentioned above, DNA damage and alterations in DNA repair are two of the main characteristics of cellular senescence (Sedelnikova et al., 2004) and are associated with aging (Brosh and Bohr, 2007). In AD, enhanced DNA damage and reduced DDR has also been demonstrated, and these alterations seem to accelerate the progression of the disease (Lovell et al., 1999).

Telomere attrition and damage have been proposed to be a potential contributor in the pathogenesis of several neurological disorders including AD (Eitan et al., 2014; Boccardi et al., 2015; Forero et al., 2016). However, there are contradictory reports on the role of telomeres alterations on AD neuropathology.

Some studies have shown that telomere shortening or alteration might be implicated in AD pathology (Aβ burden, hyperphosphorylation of tau and dementia) (Grodstein et al., 2008; Jenkins et al., 2008; Guan et al., 2012).

In monocytes of AD patients, shorter telomeres were found (Hochstrasser et al., 2012). Shorter telomere length was also found in patients with AD than in those with mild cognitive impairment than in healthy controls (Scarabino et al., 2017). Moreover, a meta-analysis of 13 studies concluded that there is evidence that AD patients are more likely to present telomere attrition (Forero et al., 2016).

However, other studies did not find changes in the length of the telomeres of patients with AD (Hinterberger et al., 2017). The telomere length of tissue obtained from the cerebellum of AD patients did not differ from age-matched controls (Lukens et al., 2009). In studies that evaluated telomere length in patients with different types of dementia (Zekry et al., 2010) or with dementia of AD types, no differences were found between affected patients and controls (Takata et al., 2012; Hinterberger et al., 2017). Also, in a mouse model of AD, telomere shortening reduces Aβ burden and improves cognition (Rolyan et al., 2011), although the animals still showed enhanced DNA damage and neurodegeneration.

Therefore, more studies are necessary to clarify the role of telomere attrition in AD neuropathology.

Additionally, an association between reduced lifespan, elevated levels of DNA damage, and a prolongation of the cell cycle duration have been demonstrated (Weirich-Schwaiger et al., 1994). In AD there is also evidence that there are significant alterations of cell cycle re-entry of post-mitotic neurons (Vincent et al., 1996; Nagy, 2005; Herrup and Yang, 2007).

During senescence, the accumulated DNA errors alter cellular functions such as transcription and DNA repair (Hackett et al., 1981; Cleaver et al., 1983; Mayne, 1984; Friedberg, 1985; Klocker et al., 1985). These alterations are more evident during aging (Staiano-Coico et al., 1983; Nette et al., 1984; Dutkowski et al., 1985; Mayer et al., 1989; Roth et al., 1989). Moreover, in AD, an enhanced DDR is found in the hippocampus and lymphocytes, as demonstrated by the presence of elevated levels of the phosphorylated histone γH2AX (H2A histone family member X) (Silva et al., 2014; Siddiqui et al., 2018).

In neurodegenerative diseases, epigenetic modifications, such as alteration in DNA methylation occur early in the disease process, affect to particular genes and correlate with misfolded proteins in specific brain regions (Armstrong et al., 2019; Prasad and Jho, 2019).

AD, PD, and DS among other neurodegenerative diseases have common aberrant DNA methylation profile that mainly affects the expression of critical genes involved in various signaling pathways that are implicated in several pathological hallmarks such as presence of Aβ plaques, NFT or α-synuclein inclusions that are present in some of them (Sanchez-Mut et al., 2016; Armstrong et al., 2019; Prasad and Jho, 2019).

Among the epigenetic alterations found in AD, DNA methylation and histone modifications show evident deregulation. It has been demonstrated that in AD brains changes in DNA methylation affect to a few common gene loci which play an essential role in the formation of Aβ plaques (Lord and Cruchaga, 2014; Watson et al., 2016; Qazi et al., 2018; Armstrong et al., 2019; Esposito and Sherr, 2019; Prasad and Jho, 2019). Among them, of particular interest are the differences in methylation in different regions of the *APP* gene promoters in the brains of humans with AD and these changes have been associated with neuropathological markers of this disorder (Bradley-Whitman and Lovell, 2013). Besides, in AD brains histone modifications have been linked to the reduction in transcription of genes that are implicated in neuronal physiology and increased the transcription of genes that are normally silenced (Berson et al., 2018). The phosphorylated histone H3 presents an altered localization of the cytoplasm, while the levels of the acetylated histone H4 are decreased in AD patients (Kwon et al., 2016).

Also, chromatin organizing proteins and epigenetic regulators are mislocated in AD, affecting chromatin architecture (Winick-Ng and Rylett, 2018). Finally, in AD, other epigenetic modifications such as alterations in chromatin remodelers, phosphorylation of histones have been found (Esposito and Sherr, 2019), and might be associated with senescence.

### Proteostasis

Altered proteostasis is a phenotypic hallmark of senescent cells. The integrity of the nucleolar ribosome biogenesis machinery and the ER, as well as the correct function of the degradation machinery (ubiquitin-proteasome and lysosomal pathways), are essential for maintaining proteostasis (Labbadia and Morimoto, 2015). The perturbation of proteostasis leads to misfolded protein accumulation and proteotoxic stress (Hetz and Mollereau, 2014; Hipp et al., 2014), which also contributes to neurodegeneration. Senescent cells lose protein homeostasis due to nucleolar dysfunction, autophagy and lysosomal anomalies and alterations in UPR indicative of ER stress. The majority of neurodegenerative disorders, present impairments of degradative compartments such as lysosomes and autophagosomes.

Protein dysfunction has been associated with numerous human neurodegenerative diseases such as AD, PD, amyotrophic lateral sclerosis, spinocerebellar ataxia, and Huntington’s disease (Yurov et al., 2014). In these diseases, high levels of different toxic protein aggregates due to a loss of protein homeostasis have been found. In particular, in AD, the formation of Aβ plaques (Ow and Dunstan, 2014), which is directly associated with the expression of SASP- associated factors (Bhat et al., 2012), and of NFT, composed by hyperphosphorylated tau protein, increase with the acquisition of a senescent phenotype and at the same time both induce senescence (Zare-Shahabadi et al., 2015; Chung et al., 2018; Mendelsohn and Larrick, 2018).

These alterations have been demonstrated to contribute to the etiology and progression of AD (Ihara et al., 2012; Nixon, 2013; Hetz and Mollereau, 2014). In this disorder, the most common form of protein aggregation, in Aβ plaques (Ow and Dunstan, 2014), which is directly associated with the expression of SASP- associated factors (Bhat et al., 2012), is one of the primary triggers and consequences of cellular senescence. The other abnormal protein found in AD brains is hyperphosphorylated tau in the form of NFTs, which have been reported to induce senescence (Mendelsohn and Larrick, 2018).

## Cellular Senescence in Down Syndrome

DS, or trisomy of chromosome 21, is the primary cause of cognitive disability of genetic origin (Shin et al., 2009). These cognitive alterations are due to defects in growth and differentiation of the CNS that appear during early prenatal stages (Haydar and Reeves, 2012; Lott, 2012). DS is characterized by premature aging and early appearance (around the fourth decade of life) of neuropathology identical to the one found in sporadic AD, including amyloid plaques, NFTs, neurodegeneration, and synapse loss (Teipel and Hampel, 2006; Sabbagh et al., 2011; Cenini et al., 2012; Lott, 2012; Wilcock and Griffin, 2013; Casanova et al., 1985; McGeer et al., 1985). Both, premature aging and AD pathology are thought to be the leading causes of the earlier mortality of this population (Schupf and Sergievsky, 2002; Zigman and Lott, 2007; Prasher et al., 2008; Esbensen, 2010).

### Neuroinflammation

The DS brain also present neuroinflammatory changes typical of AD, such as microglial activation and increased release of pro-inflammatory cytokines (Griffin et al., 1989; Park et al., 2005; Griffin, 2006; Wilcock and Griffin, 2013). There is compelling evidence that, in DS, the increased release of cytokines due to microglia activation, enhances the production of ROS, aggravates synaptic dysfunction, and neurodegeneration, and reduces neurogenesis (Town et al., 2005; Fuster-Matanzo et al., 2013; Llorens-Martín et al., 2014; Lyman et al., 2014; Rosi et al., 2012). The aggravation produced by neuroinflammation on neurodegeneration and the reduced neurogenesis (see Rueda et al., 2012), render DS individuals more susceptible to neuropathological events that can accelerate the onset of dementia (Teipel and Hampel, 2006).

Enhanced microglial activation also produces an increased expression of proinflammatory cytokines in different areas of the brain of a mouse model of DS (Hunter et al., 2004; Lockrow et al., 2011; Roberson et al., 2012; Rueda et al., 2018). Reducing neuroinflammation by administering an antibody against the pro-inflammatory cytokine IL17A to these mice, reduces cellular senescence in the hippocampus and cortex of these animals, improves their cognitive abilities and reduces some of the signs of neurodegeneration (Rueda et al., 2018). These results suggest a direct link between cellular senescence, neuroinflammation and neurodegeneration in DS.

### Oxidative Stress and Mitochondrial Dysfunction

Another crucial mechanism that contributes to accelerating aging and cognitive dysfunction in DS is oxidative stress. In this syndrome, it is present from embryonic stages producing a reduction in neuronal proliferation, differentiation, connectivity, and survival (Monti et al., 1992; Busciglio and Yankner, 1995; Perluigi et al., 2011). As the person with DS ages, the levels of oxidative stress increases, contributing to the progression of cognitive and neuronal degeneration (Busciglio et al., 2007; Lockrow et al., 2009; Shichiri et al., 2011; Perluigi and Butterfield, 2012; Rueda et al., 2012; Parisotto et al., 2016). As previously mentioned, oxidative stress is an essential factor that causes cellular senescence (Monti et al., 1992; He et al., 2013; Rodríguez-Sureda et al., 2015).

Furthermore, mitochondrial dysfunction is also present in DS cells during embryonic life (Perluigi et al., 2011), leading to profound alterations in energy metabolism due to the reduced synthesis of ATP synthesis (Valenti et al., 2010). ATP is also reduced *in vitro* in DS fibroblasts, which could contribute to premature cell aging (Rodríguez-Sureda et al., 2015).

In DS, early induction of senescence by aberrant intracellular oxidant activity and antioxidant defense has been demonstrated in fibroblasts, skin tissue, lymphocytes (De Haan et al., 1996; Kalanj-Bognar et al., 2002; Cristofalo et al., 2004; Kimura et al., 2005; Biron-Shental et al., 2015; Rodríguez-Sureda et al., 2015) and in the amniotic fluid of women carrying DS fetuses (Perluigi et al., 2011; Amiel et al., 2013). Thus, senescence is present in DS from prenatal stages and can be responsible for different altered phenotypes. But, enhanced senescence is a prominent phenomenon during all the life stages of a DS individual and is aggravated with aging and the appearance of AD neuropathology.

In agreement with these results, it has been demonstrated that the hippocampus of a mouse model of DS shows higher amounts of oxidative damage (protein and lipid oxidative damage), Aβ expression and tau phosphorylation, as well as increased density of senescent cells in different hippocampal regions (i.e., DG, CA1, CA3, and hilus; Corrales et al., 2013; Parisotto et al., 2016; García-Cerro et al., 2017; Rueda et al., 2018). Other authors have also found the early presence of SA-β-gal activity in both cultured fibroblasts and skin tissue from DS mice (Contestabile et al., 2009b). Finally, chronic administration of melatonin, a potent antioxidant, to this mouse model of DS reduces hippocampal senescence and oxidative damage, improves the cognitive abilities of these mice, enhances neurogenesis and reduces their neurodegenerative phenotype (Corrales et al., 2013, 2014; Parisotto et al., 2016). These results provide support for the association between oxidative stress and senescence in DS.

### DNA Damage and Repair

Hematopoietic stem cells and satellite cells of skeletal myofibers in the Ts65Dn mouse model of DS accumulate DNA damage and prematurely develop a senescent phenotype (Adorno et al., 2013; Wang et al., 2016; Pawlikowski et al., 2018). Furthermore, in this murine model, the ubiquitin-specific-peptidase 16 (*Usp16*) gene is triplicated, leading to the overexpression of the Usp16 enzyme that controls the ubiquitination state of the histone 2A (Ub-H2A) and contributes to regulate the DDR chromatin remodeling and cell cycle progression (Joo et al., 2007). Thus, in these mice, both the overexpression of the *Usp16* gene, which may affect the DNA damage/repair signaling pathways and the high levels of brain oxidative stress and neuroinflammation may produce and excessive accumulation of DNA damage leading to genomic instability and neurodegeneration or premature cellular senescence.

In DS, there is also evidence of prenatal accumulation of DNA damage and of the reduction of the ability to repair DNA (Agarwal et al., 1970; Raji et al., 1998; Pogribna et al., 2001; Cabelof et al., 2009; Nižetić and Groet, 2012; Di Domenico et al., 2015). Besides, in response to DNA damage, DNA polymerase b (POLb), whose function is to participate in base excision repair (BER) is induced; however, in DS fibroblasts its expression is reduced, thereby reducing the ability to repair DNA damage. This reduction is sufficient to induce senescence (Ahmed et al., 2018; Cabelof et al., 2002, 2003; Cabelof, 2007).

In DS, both alterations in the DNA damage/repair signaling pathways, and the high levels of brain oxidative stress may produce and excessive accumulation of DNA damage leading to genomic instability and neurodegeneration or premature cellular senescence. Hence, this mechanism might play an essential role in the onset and aggravation of the AD-associated cognitive decline.

DNA repair mechanisms eliminate damages and elongate cell cycle duration to give the repair enzymes time to work. However, alterations in DNA repair mechanisms producing accumulated DNA damage can delay the cell cycle until mitotic activity ceases, leading to premature senescence and sometimes cell death. In DS, enhanced DNA damage and impairment in DNA repair impairs mitosis and delays the cell cycle leading to senescence. There is evidence of delayed cell cycle in DS individuals and mouse models of this syndrome (Contestabile et al., 2007, 2009a).

As mentioned above, telomere attrition is one of the best-characterized senescence-triggering mechanisms. Telomeres are shorter in DS amniocytes, and placentas of fetuses with DS present an increased percentage of trophoblasts with senescence phenotypes (Sukenik-Halevy et al., 2011; Amiel et al., 2013; Biron-Shental et al., 2015). For this reason, it has been suggested that it could be an early biomarker of premature senescence (Vaziri et al., 1993). Also, a significantly higher loss of telomere sequences was observed *in vitro* and *in vivo* in lymphocytes from DS individuals (0–45 years old) (Vaziri et al., 1993; de Arruda Cardoso Smith et al., 2004). Jenkins et al. (2008) also demonstrated that shorter telomere length correlates with the degree of dementia and AD-associated neurodegeneration in DS individuals. These results suggest that senescence can play an essential role in the aging of the immune system in DS (Vaziri et al., 1993), leading to the altered microglia function and neuroinflammation. Thus, in DS, this type of DNA damage can induce senescence from prenatal stages through the entire lifespan of the individual.

### Epigenetic Modifications

Epigenetic alterations such as DNA and histone modifications are also found in patients with DS. Among them, DNA hypermethylation, histone modifications, histone core variants and changes in small non-coding RNAs are implicated in several phenotypic characteristics of DS such as memory impairment, premature aging and neurodevelopmental defects (Mentis, 2016).

Although some of these alterations are associated with premature aging in DS, the exact implication of epigenetic modifications in cellular senescence found in this syndrome needs to be elucidated.

### Proteostasis

Abnormalities in chromosome number disrupt proteostasis (Oromendia et al., 2012; Oromendia and Amon, 2014; Stingele et al., 2012). Gene copy number changes due to aneuploidy can affect the number of proteins expressed, their functions and the protein quality-control and repair machinery of the cell (Sheltzer et al., 2011; Oromendia et al., 2012; Thorburn et al., 2013; Yurov et al., 2014).

Thus, in DS, the extra chromosome can alter proteostasis (Lockstone et al., 2007). Trisomy may also affect the 3D nuclear architecture of the genome resulting in changes in gene interactions and gene expression that may impact proteostasis regulation (Vermunt et al., 2019).

Regarding protein synthesis, only a few studies have analyzed the ribosome biogenesis machinery in DS. Demirtas (2009), using a classic silver staining procedure to study the arrangement of the nucleolar organizer regions (NORs), found that cells from DS infants presented an increased number of AgNOR dots. Because ribosome biogenesis is a highly energy-consuming process, these authors suggest that this response to trisomy 21 might result in unnecessary rRNA and ribosomal protein synthesis, which leads to a waste in energy. Also, reduced expression of specific transcription, splicing, and translation factors has been found in cortices of fetuses with DS (Freidl et al., 2001), suggesting a deranged protein synthesis in them.

Moreover, in DS, both intracellular degradative systems, UPS and autophagy, seem to be affected. Chronic exposure to oxidative stress, as occurs in the DS brain, also causes protein oxidation of members of the proteostasis network, resulting in accumulation of unfolded/damaged protein aggregates and dysfunction of intracellular degradative system, such as autophagy and the ubiquitin-proteasome systems (UPS), contributing to neurodegeneration (Di Domenico et al., 2013; Hetz and Mollereau, 2014). In the hippocampus of Ts65Dn mice altered autophagy, due to reduction of autophagosome formation, has been found (Tramutola et al., 2016). In DS human fibroblasts and in the brain of two segmental trisomy models of DS, the Dp16 and Dp17 mice, increased ubiquitination, and disrupted proteasome activity has been reported (Aivazidis et al., 2017). Finally, in the cerebellum of the Ts65Dn mouse, a dysfunction of the UPS, due to reduced proteasome activity and a parallel increase of ubiquitinated proteins, has been implicated in the degeneration of Purkinje cells (Necchi et al., 2011). Consequently, ubiquitinated or polyubiquitinated proteins are not degraded at a normal rate and are stored in the nuclei of cerebellar neurons of this model (Necchi et al., 2011).

Besides, disruption in the proteostasis network could contribute to the accumulation of protein aggregates, such as amyloid deposits and NFTs (Di Domenico et al., 2013). Alterations of the proteostasis network are present in individuals with DS years before the age-related cognitive decline, and AD-associated dementia is detected.

In summary, DS is characterized by early senescence that continues throughout the lifespan of the individual and is very likely aggravated by the appearance of AD neuropathology.

## Cellular Senescence in Parkinson Disease

PD is a progressive neurodegenerative disorder characterized by the loss of dopaminergic neurons in the substantia nigra, the presence of cytoplasmic protein aggregates, known as Lewy bodies, that contain a variety of proteins, including ubiquitin and α-synuclein (Nussbaum and Ellis, 2003; Thomas and Beal, 2011). These pathological features result in an impairment of motor control, including difficulty to initiate movements, loss of balance, rigidity, and tremor, and cognitive deterioration (Poewe et al., 2017). The most important risk factors for the development of PD are aging, a genetic predisposition and exposure to toxins (Chinta et al., 2013).

Similarly to AD and DS, PD neuropathology is aggravated by neuroinflammation mitochondrial dysfunction, α-synuclein accumulation, oxidative stress, and cellular senescence (Poewe et al., 2017).

There is widespread evidence that cellular senescence plays an essential role in the pathogenesis of PD. Patients with PD present enhanced levels of SA-β-gal in their CSF (van Dijk et al., 2013) and in their brain tissue (Chinta et al., 2018). Higher numbers of senescent astrocytes are also present in the substantia nigra of patients with PD (Chinta et al., 2018). Besides, paraquat, a herbicide that has been proposed to be implicated in the appearance of some types of PD, induces senescence in human astrocytes (Chinta et al., 2018).

### Neuroinflammation

Similarly to AD and DS, inflammation is prominent in PD (Chinta et al., 2018). Activation of microglia is thought to be one of the main determinants of dopaminergic loss in the substantia nigra (McGeer et al., 1988), hippocampus, cingulate and temporal cortex (Imamura et al., 2003). α-synuclein aggregation activates microglial cells in PD (Zhang et al., 2005). The result of microglia activation is in the increased levels of the pro-inflammatory mediators IL-1, IL-6, TNFγ and TNF-α in the CSF, serum, and dopaminergic regions of the striatum from patients with PD (Mogi et al., 1994a,b; Blum-Degen et al., 1995; McCoy et al., 2006; Mount et al., 2007; Brodacki et al., 2008; Scalzo et al., 2010; Lindqvist et al., 2012; Dursun et al., 2015).

The high presence of senescent cells and aged astrocytes in PDs brains suggests that senescence-induced neuroinflammation might be an important mechanism for PD neurodegeneration and a potential therapeutic target for this disease.

### Oxidative Stress and Mitochondrial Dysfunction

Oxidative stress plays an essential role in the etiology and progression of PD (Trist et al., 2019). Even in the early stages of the disease, before a significant loss of dopaminergic neurons, PD patients present elevated oxidative stress (Ferrer et al., 2011). Thus, oxidative stress could be partially responsible for neurodegeneration, in addition to been enhanced as a result of the neuronal loss. As demonstrated in AD and DS, it seems that positive feedback is produced between both insults that probably aggravates the disease.

Mitochondrial dysfunction also plays a vital role in PD etiopathology. In familial PD, different mutations in the genes associated with pathways of mitochondrial dysfunction have been demonstrated, and some of these pathways are also altered in sporadic PD (Park et al., 2018). The production of ROS during ATP synthesis is higher in dopaminergic neurons of the substantia nigra. Due to their size and complexity, they need more elevated amounts of ATP to maintain resting membrane potential, propagate action potentials, and enable synaptic transmission (Pissadaki and Bolam, 2013).

These alterations in mitochondrial function and oxidative stress are likely to be a result of senescence.

### DNA Damage and Repair

DNA damage and repair have been proposed as important actors altering the function of the dopaminergic system in PD (Sepe et al., 2016). Telomere shortening plays a causal role in cellular aging (Hastie et al., 1990), and it has been proposed as a potential contributor in the pathogenesis of PD (Anitha et al., 2019).

The studies of telomere length in PD patients had provided contradictory results. Some of them provide support for shorter telomere length in different cell types or tissues (Tomac and Hoffer, 2001; Guan et al., 2008; Koliada et al., 2014) while others do not find evidence of this alteration in PD (Guan et al., 2008; Wang et al., 2008; Eerola et al., 2010; Watfa et al., 2011; Maeda et al., 2012; Degerman et al., 2014; Eitan et al., 2014; Forero et al., 2016). A meta-analysis of all these studies concluded that PD patients do not present changes in telomere length (Forero et al., 2016). Thus, new studies are necessary to clarify the putative role of replicative senescence and telomere shortening in different brain regions and cell types in PD.

However, it has been demonstrated that PD is characterized by genetic instability due to gene mutations, altered gene expression, or regulation. The expression of some genes implicated in the cell cycle, such as *p16INK4a* (Chinta et al., 2018) and *pRb* (Ihara et al., 2012), are upregulated in PD brains. This enhanced genetic expression is likely to produce alterations in the cell cycle in this disorder.

Furthermore, alterations in epigenetic modulation seem to play an important role in the etiopathology of PD. The brains of these patients present changes in DNA methylation, post-translational modifications of histones and of microRNAs that regulate pathways involved in the pathophysiology of PD (Armstrong et al., 2019; Prasad and Jho, 2019; Renani et al., 2019).

However, the role of the alterations of DNA methylation in the pathological hallmarks of this disease, including cellular senescence, is still unclear.

### Proteostasis

Another characteristic that PD shares with other neurodegenerative diseases is altered proteostasis. In PD, there is an accumulation of misfolded proteins in the brain (Martinez et al., 2019). All patients with PD present insoluble α-synuclein fibrils, which damage neurons, and are associated with intracellular Lewy bodies (Melki, 2015). Alterations in the proteostasis network seem to play a prominent role in the protein aggregation found in PD, mainly due to changes in the function of the ER, the organelle involved in protein folding and quality control. ER stress produces the UPR to try to restore proteostasis.

In this disease, the increase in senescent cells is thought to be directly associated with the enhanced aggregation of α-synuclein (Chinta et al., 2018).

It has been proposed that mitochondrial dysfunction and oxidative stress could be partially responsible for this α- synuclein accumulation (Dias et al., 2013; Rocha et al., 2018).

Finally, the autophagic/lysosomal dysfunction also plays an essential role in the pathogenesis of the disease, since different genetic mutations that produce defects in these pathways are found in PD patients (Rivero-Ríos et al., 2016; Pitcairn et al., 2018).

In summary, cellular senescence is also a pathological event of crucial importance in PD, and shares with other neurodegenerative diseases many of the factors that trigger it and aggravate the neuropathology (i.e., oxidative stress, mitochondrial dysfunction, altered proteostasis, among others).

## Concluding Remarks

Senescent cells accumulate with aging and in neurodegenerative pathologies, and there is extensive evidence that they might be implicated in the etiopathology of these diseases. Here, we reviewed the data that demonstrate that cellular senescence is a common pathological feature of PD, AD, and DS. These three diseases also share some of the mechanisms that promote senescence and that allow this permanent state of cell arrest to induce or aggravate neurodegeneration. Furthermore, extensive senescence has also been found in other neurodegenerative diseases such as Huntington Disease, Multiple Sclerosis, and Rett syndrome. Thus, senescence seems to be a common mechanism for neurodegeneration that needs to be further investigated.

At present, there are no efficient neuroprotective treatments that can prevent or delay the progression of the disease for AD in individuals with or without DS or for PD. Thus, new neuroprotective therapeutic approaches are needed.

Because of the central role of cellular senescence in the etiopathology of different neurodegenerative diseases, therapies that reduce senescence could be a promising approach to prevent the loss of cells and the alteration of their function. These therapies have been more extensively investigated in the case of AD (He et al., 2013; Kirkland and Tchkonia, 2017).

Several therapeutic strategies, known as senotherapies, to selective eliminate tissue-specific senescent cells, have been developed (Childs et al., 2017; Kritsilis et al., 2018; Kim and Kim, 2019). However, almost none have been tested in models of different neurodegenerative diseases, except AD. Clearance of senescent cells extended lifespan in normal mice (Baker et al., 2016) and in a progeroid mouse model (Baker et al., 2011). Finally, a recent work has demonstrated that pharmacological elimination of senescent astrocytes and microglia cells using a senolytic compound (ABT263) attenuates tau phosphorylation, modulates tau aggregation, prevents the upregulation of senescence-associated genes in the cortex and hippocampus and improves short-term memory in a transgenic mouse model that overexpresses human tau (Kirkland and Tchkonia, 2017; Bussian et al., 2018). Thus, senotherapeutic agents may serve as powerful tools to prevent neurodegenerative diseases.

Importantly, in many of these disorders, senescence is an early event that appears years before other signs of neurodegeneration and in the case of DS, even at embryonic stages, providing a broad therapeutic window to prevent or delay some of the pathological processes associated.

Finally, many of the mechanisms implicated in the onset of senescence and that also appear years earlier than neurodegeneration (i.e., oxidative stress, mitochondrial damage, neuroinflammation, DNA damage, altered proteostasis), are also a result of senescence, therefore aggravating the disease (Figure 1). Thus, therapies targeting these earlier events could exert a double benefit preventing the onset of the neurodegenerative disease or delaying its progress.

## Author Contributions

CM-C and NR wrote and edited the manuscript.

## Conflict of Interest

The authors declare that the research was conducted in the absence of any commercial or financial relationships that could be construed as a potential conflict of interest.
